# Supplementary material for: Content-rich biological network constructed by mining PubMed abstracts
Source: BMC Bioinformatics. 2004 Oct 8;5:147. doi: 10.1186/1471-2105-5-147 (PMC528731; doi:10.1186/1471-2105-5-147)
Supplement: Additional File 2 — The original results of the above study (non-essential files are deleted to keep the file size under the limit set by BMC bioinformatics). [file 1471-2105-5-147-S2.bz2 › chilibotAdditionalFile2/dip10/403ID8955093E10063/html/IL6ST_OSM.html]

 


 **IL6ST** and **OSM** 
  
Found 243 abstracts in PubMed, retrieved 10.  
 

 What does Google say? 
 PDF only 
| .edu only 

---

**Interactive relationship** (e.g. stimulation, inhibition, etc)

**Inhibitory relationship**- Desensitization of signaling by oncostatin M  [ **OSM** ]  in human vascular cells involves cytoplasmic Tyr residue 759 in gp130  [ **IL6ST** ]  but is not mediated by either Src homology 2 domain containing tyrosine phosphatase 2 or suppressor of cytokine signaling 3.  Ref: 12724316 J Biol Chem< MedlineTA>J Biol Chem,
- The leukemia inhibitory factor LIF, cardiotrophin 1 CT 1 and oncostatin M  [ **OSM** ]   **OSM**  are four helix bundle cytokines acting through a common heterodimeric receptor composed of gp130  [ **IL6ST** ]  and LIF receptor LIFR.  Ref: 12707269 J Biol Chem< MedlineTA>J Biol Chem,
**Neutral relationship**- Oncostatin M  [ **OSM** ]  OnM signals through cell surface receptors which utilize the gp130  [ **IL6ST** ]  subunit.  Ref: 12724316 J Biol Chem< MedlineTA>J Biol Chem,
- Here we report the purification and characterization of one of these down regulatory factors, the cytokine, oncostatin M  [ **OSM** ]   **OSM** , which transmits its signal via the gp130  [ **IL6ST** ]  cell surface receptor, resulting in the selective down modulation of the melanocyte lineage antigens Melan A MART 1, gp100, tyrosinase, tyrosinase related proteins 1 and 2, and the M isoform of microphthalmia transcription factor.  Ref: 12692260 Mol Cancer Res, 2003

**Non-interactive relationship** (e.g. studied together, co-existance, homology, etc.)

- To effect synergy on the PGE 2 level,  **OSM**  signals in part through its gp130  [ **IL6ST** ]  OSMRbeta receptor, since neutralizing antibodies against gp130  [ **IL6ST** ]  and OSMRbeta, but not LIFRbeta, decrease PGE 2 production in response to  **OSM**  plus IL 1beta.  Ref: 12730964 Glia, 2003
- Localization of oncostatin M  [ **OSM** ]  receptor  [ **IL6ST** ]  beta in adult and developing CNS.  Ref: 12831858 Neuroscience, 2003
- Our study demonstrated that beta subunit of oncostatin M  [ **OSM** ]  receptor  [ **IL6ST** ]  was expressed in both cell bodies and processes of nonpeptidergic nociceptive neurons in adult mice, suggesting that oncostatin M  [ **OSM** ]  may affect the nociceptive function of the neurons through the modulation of vanilloid receptor 1 and P2X3 expression.  Ref: 12814362 Eur J Neurosci, 2003
- Here, we investigated the expression of specific beta subunit of oncostatin M  [ **OSM** ]  receptor  [ **IL6ST** ]  in the dorsal root ganglia of adult mice.  Ref: 12814362 Eur J Neurosci, 2003
- Seven days after sciatic nerve axotomy, the expression of beta subunit of oncostatin M  [ **OSM** ]  receptor  [ **IL6ST** ]  was down regulated in the lumbar dorsal root ganglia of the injured side.  Ref: 12814362 Eur J Neurosci, 2003
- Expression of oncostatin M  [ **OSM** ]  receptor  [ **IL6ST** ]  beta in a specific subset of nociceptive sensory neurons.  Ref: 12814362 Eur J Neurosci, 2003
- Targeted disruption of Oncostatin M  [ **OSM** ]  receptor  [ **IL6ST** ]  results in altered hematopoiesis.  Ref: 12855584 Blood< MedlineTA>Blood, 2003
- Reverse transcription PCR analysis of ESCC and matched normal esophageal tissues provided the first evidence that melanoma metastasis clone D MEMD, a gene similar to oncostatin M  [ **OSM** ]  receptor  [ **IL6ST** ]  beta, and KIAA0471 gene products are upregulated in ESCCs.  Ref: 12706106 Genomics, 2003
- In the adult dorsal root ganglia, beta subunit of oncostatin M  [ **OSM** ]  receptor  [ **IL6ST** ]  was exclusively expressed in small sized neurons.  Ref: 12814362 Eur J Neurosci, 2003
- In addition, all beta subunit of oncostatin M  [ **OSM** ]  receptor  [ **IL6ST** ]  positive neurons expressed both vanilloid receptor 1 and P2X3 purinergic receptor.  Ref: 12814362 Eur J Neurosci, 2003
- Approximately 13% of total dorsal root ganglia neurons in mice contained beta subunit of oncostatin M  [ **OSM** ]  receptor  [ **IL6ST** ] .  Ref: 12814362 Eur J Neurosci, 2003
- The double immunofluorescence method revealed that approximately 28% of beta subunit of oncostatin M  [ **OSM** ]  receptor  [ **IL6ST** ]  positive neurons contained TrkA immunoreactivity, 63% expressed Ret immunoreactivity and 58% bound isolectin B4.  Ref: 12814362 Eur J Neurosci, 2003
- IL 6 type cytokines exert their action via the signal transducers gp130  [ **IL6ST** ] , LIF receptor and  **OSM**  receptor leading to the activation of the JAK STAT and MAPK cascades.This review focuses on recent progress in the understanding of the molecular mechanisms of IL 6 type cytokine signal transduction.  Ref: 12773095 Biochem J< MedlineTA>Biochem J, 2003
